# Déterminants de l'utilisation des méthodes contraceptives dans la zone de santé Mumbunda à Lubumbashi, République Démocratique du Congo

**DOI:** 10.11604/pamj.2015.22.329.6262

**Published:** 2015-12-03

**Authors:** Charles Matungulu Matungulu, Simon Ilunga Kandolo, Abel Ntambue Mukengeshayi, Angèle Musau Nkola, Dorcas Ilunga Mpoyi, Sylvie Katanga Mumba, Julie Ndayi Kabamba, Karen Cowgill, Françoise Malonga Kaj

**Affiliations:** 1 Ecole de Santé Publique de l'Université de Lubumbashi, RD Congo; 2 Seattle University college of Nursing, USA

**Keywords:** Déterminant, planification familiale, méthodes contraceptives, contraception, Zone de santé, Lubumbashi, Determinants, family planning, contraceptive methods, contraception, health zone, Lubumbashi

## Abstract

**Introduction:**

Augmenter la prévalence contraceptive s'incarne dans les objectifs des tous les acteurs des programmes qui visent de réduire la mortalité maternelle et infantile, d'améliorer la santé reproductive des adolescents, de lutter contre le VIH/SIDA et les infections sexuellement transmissible (IST), de promouvoir le bien-être familial et de ralentir la croissance démographique. L'objectif de cette étude était de déterminer la prévalence contraceptive moderne et identifier les facteurs qui sont liés à l'utilisation des méthodes contraceptives dans la zone de santé de Mumbunda.

**Méthodes:**

Une étude transversale à visé analytique a été effectuée auprès des femmes âgées de 15 à 49 ans en union maritale, de Mai à Juin 2014. Grâce à un questionnaire pré testé et validé, nous avons récolté les données par interview sur les caractéristiques sociodémographiques, obstétricales ainsi que sur la pratique contraceptive. Le logiciel SPSS version 21 nous a permis d'analyser les données.

**Résultats:**

Au total 500 femmes ont été incluses dans cette étude dont l’âge moyen était de 27,9±6,1 ans. La prévalence contraceptive moderne était de 27,6%. L'attitude (ORa= 4,79; IC95%: 1,59-14,43; p<0,001), le niveau de connaissance des méthodes contraceptives (ORa=1,87; IC95%: 1,22-2,87; p<0,001), le soutien du conjoint (ORa=1,87; IC95%: 1,22-2,87; p<0,001) étaient significativement associés à l'utilisation des méthodes contraceptives modernes.

**Conclusion:**

Tout effort d'augmentation de la prévalence contraceptive devrait cibler l'attitude, le niveau de connaissance de méthodes et le soutien du conjoint afin d'optimiser l'utilisation de la contraception moderne dans la zone de santé (ZS) Mumbunda.

## Introduction

La planification familiale, est l'une des composantes essentielle des soins de santé primaire et de santé de la reproduction, visant à améliorer la santé de la mère, du nouveau-né et de l'enfant, par la réduction de la morbidité et de la mortalité dans ces catégorie, ainsi que la transmission du VIH/SIDA [1]. La contraception pourrait empêcher à environ 104 000 décès maternel chaque année, soit une réduction de 29% [2]. Elle peut réduire la mortalité juvénile de près de 10%, si elle est disponible à tous ceux qui en ont besoin [3]. La République Démocratique du Congo (RD Congo), comme beaucoup de pays de l'Afrique Subsaharienne, se caractérise par un faible taux d'utilisation de méthodes contraceptives modernes, bien qu'il soit parmi les pays caractérisés par des taux très élevés de mortalité maternelle et infantile [2]. Le ratio de mortalité maternelle est estimé à 549 pour 100 000 naissances vivantes; donc une femme sur 29 court un risque de décéder pour cause maternelle pendant les âges de procréation [4]. Les chiffres récents sur la mortalité infantile font état de 97 décès pour 1000 naissances vivantes [5]; le même rapport donne le taux de mortalité infanto-juvénile de 158 pour 1000 naissances vivantes. L'indice synthétique de fécondité se chiffre à 6,6 enfants par femme pour les milieux rural et urbain et 5,4 enfants dans le milieu urbain [6]. Cela montre l'absence du contrôle de la fécondité selon ce que postule Henry, cité dans le rapport des enquêtes démographique et sanitaire de la RD Congo (EDS-RDC) de 2007 [4].

Depuis 1970, la RD Congo se caractérise par un taux de croissance parmi les plus élevés au monde, soit plus de 3% [4]. La croissance rapide de la population (>2%) et une fécondité élevée, sont une menace pour le bien être des individus et des sociétés les plus pauvres dans les pays en voie de développement [7]. En RD Congo, sept ménages sur dix sont pauvres [8]. La prévalence contraceptive moderne a été de 5,4% en 2010 [5]. Les résultats des enquêtes démographiques et sanitaires de 2013 font état d'une prévalence de 8% pour l'ensemble du pays, et de 15% dans le milieu urbain contre 5% dans le milieu rural [6]. Cela témoigne d'un progrès très faible. De ce fait, le gouvernement national s'est engagé à augmenter la prévalence contraceptive moderne à 19% dans l'ensemble du pays, sur une échéance allant de 2014 à 2020 [9]. Ce même document révèle que la couverture en activités de planification familiale est faible dans le pays. La réalisation de cet objectif, devra se concrétiser dans chaque Zone de Santé, en sa qualité d'unité opérationnelle de la mise en œuvre de la politique nationale de santé, au sein du système de santé de la RD Congo [10]. La zone de santé Mumbunda fait partie du district sanitaire de Lubumbashi, dans la ville portant le même nom, deuxième ville du pays après la capitale. Elle se caractérise par une absence des données sur l'ensemble des femmes en âge de procréer en matière de planification familiale. Aucune source d'information ne peut renseigner ni sur la prévalence contraceptive locale, ni sur les déterminants de l'utilisation des méthodes contraceptives modernes.**Objectifs**: déterminer la prévalence contraceptive moderne et identifier les facteurs qui sont associés à l'utilisation des méthodes contraceptives dans la zone de santé de Mumbunda.

## Méthodes

Nous avons mené une étude transversale à visé analytique de Mais à Juin 2014. Elle a concerné toutes les femmes en âge de procréer (de 15 à 49 ans) en union de la Zone de Santé de Mumbunda, d'où un échantillon a été tiré grâce à la formule suivante: n=z_2_pq/d_2_.

L’échantillonnage en grappe nous a permis de sélectionner les ménages, à l'intérieur des quels, les femmes âgées de 15 à 49 ans étaient interrogées. Les avenues ont été considérées comme grappes, dont l'effectif s’élevait à 59. La sélection de ces avenues était effectuée par l'aléatoire simple, en se servant des croquis des aires de santé pour leur identification. Grâce à un questionnaire pretesté et validé, nous avions récolté des données par interview. Une équipe d'enquêteurs était recrutée et formée avant la descente dans les ménages afin d'administrer le questionnaire qui était composé des parties suivantes: caractéristiques des ménages et du logement, identité de la femme et du conjoint, fécondité, information sur les méthodes contraceptives, connaissance de méthodes, utilisation des méthodes contraceptives.

### Analyse des données

La partie descriptive a consisté à décrire les caractéristiques socio démographiques des nos répondantes, antécédents obstétricaux, connaissance des méthodes, utilisation et non utilisation de la contraception; cette description a généré les pourcentages, la moyenne et son écart type. La partie analytique a quant à elle, a consisté en la recherche des associations entre l'utilisation des méthodes contraceptives et les facteurs prédicteurs de l'utilisation des méthodes modernes: c'est l'analyse uni variée. Pour ce faire, le test de Khi carré de PEARSON, au seuil de risque de 5% a été utilisé, ainsi que le test de Fisher exact selon les conditions usuelles de chaque test [11]. Cette étape était déterminante pour choisir les variables à entrer dans le modèle sous condition d'indépendance de chacune des ces variables. A ce stade, si deux variables étaient liées, l'une d'entre elles était retenue pour la régression logistique. Pout toutes les variables retenues, le rapport des côtes brut ou Odd ratio brut (RC ou OR) a été calculé, en conformité avec ses conditions d'usage [11]. Pour toutes les variables, la modalité de référence (non exposé) était définie en référence avec la revue de la littérature, comme on le verra dans le tableau croisé. En plus toutes les variables ont été dichotomisées (variable binaire) selon les exigences de la régression logistique. Ainsi le chiffre zéro (0) correspondait au non usage des méthodes moderne de contraception et le chiffre un (1) l'utilisation de ces méthodes. La régression logistique nous a permis de générer le modèle des déterminants de l'utilisation des méthodes contraceptives, par le calcul des OR ajustés. Les variables à inclure dans le modèle ont été sélectionnées selon la méthode ascendante de Wald, qui est une méthode de sélection pas à pas avec test d´entrée fondé sur la signification de la statistique de significativité (avec p<0,2) et avec test de suppression fondé sur la probabilité de la statistique de Wald. L’équation de la régression se présente comme suit:

P(Y=1/X_1_, X_2_,…, X_k_)=1/(1+e(β_0_+Σ^k^_i=1_β_i_X_i_))

La vérification de la qualité de l'ajustement a été effectuée grâce au test de Hosmer-Lemeshow. Seules les variables qui ont un effet statistiquement significatif sur l'utilisation des méthodes contraceptives modernes ont été retenues dans le modèle final.

### Variable dépendante

Nous avons considéré dans le cadre de la présente étude, l'utilisation des méthodes contraceptives modernes comme variable dépendante, qui est dichotomique: utilisation (oui=1) et non utilisation (Non=0). Par méthode contraceptive moderne, nous avons considéré les méthodes suivantes: préservatif, pilule, injectables (depo provera), implant, dispositif intra utérin (DIU) car elles ont été les seules à être citées par les femmes. Par non utilisation des méthodes contraceptives modernes, nous avons considéré uniquement les femmes qui avaient l'information sur l'existence de ces méthodes et qui n'en faisaient pas usage, sous l'hypothèse que celles qui n’étaient pas informées, elles les utiliseraient; d'où leur exclusion car cette hypothèse est difficile à être vérifier dans cette étude.

### Variables indépendantes

Nous avons retenu les facteurs prédicteurs de l'utilisation de la contraception suivants: âge, statut matrimonial, niveau d’étude, religion, occupation de la femme, niveau de confort, nombre d'enfants vivants, attitude de la femme face aux méthodes, niveau de connaissance des méthodes contraceptives, discussion avec le conjoint, soutien du conjoint et le niveau d’étude du conjoint. Nous avons utilisé le logiciel Excel pour encoder les données et Epi Info version 3.3.2 pour effectuer les analyses uni variées. Le logiciel SPSS version 21 nous a permis de réaliser la régression logistique en générant les OR ajustés. Les tests statistiques suivants ont été utilisés: l'Odd Ratio brut et ajusté et son intervalle de confiance à 95%, le Khi carré au seuil de risque d'erreur à 5% et le Fisher exact au même seuil de signification.

## Résultats

L’âge moyen des femmes était de 27,9±6,1 ans (Min: 17 ans; Max: 49 ans), la gestité moyenne était de 2,9±1,9 grossesses (Min: 0; Max: 12), la parité moyenne était de 2,8±1,7 (Min: 0; Max: 9); 91% des femmes étaient informées sur les méthodes contraceptives (Tableau 1) et les formations sanitaires étaient la source importante de cette information (75,6%) (Tableau 2). Toutes (100%) les femmes informées sur la contraception connaissaient au moins une méthode contraceptive (moderne et/ou traditionnelle). Nous avons observé que la prévalence du préservatif masculin était de 17,6%, celle de la pilule était de 5%, celle depo provera s'elevait à 2, 4%, le DIU (dispositif intra utérin) n’était utilisé que par 1% de femmes et l'implan par 1,6% (Tableau 3). La prévalence contraceptive moderne était de 27,6% et celle des méthodes naturelle s’élève à 36,8% (Tableau 4). L'analyse multi variée (Tableau 5, Tableau 6, Tableau 7) nous a montré que les femmes qui avaient une attitude favorable aux méthodes contraceptives, avaient dix fois plus de chance d’être utilisatrices des méthodes modernes de contraception que celles ayant une attitude défavorable (OR=10,38; IC95%: 3,55- 34,14); même après ajustement sur les autres variables explicatives, cette association a été présente, (ORa= 4,79; IC95%: 1,59-14,43). Les femmes qui avaient un niveau élevé de connaissance des méthodes contraceptives, avaient près de deux fois plus la chance d’être utilisatrices que celles ayant un niveau faible de connaissance (OR=1,93; IC95%: 1,26-2,95); après ajustement sur les autres variables, la chance ne varie quasiment pas (ORa=1,87, IC95%: 1,22-2,87). Nous avons également observé que les femmes qui discutaient couramment et rarement avec leurs partenaires, avaient respectivement six (OR=6,23; IC: 2,91-13, 69) et trois (OR= 2,67; IC95%= 1,22- 6,00) fois plus la chance d'utiliser les contraceptifs modernes que celles qui ne discutaient jamais. Mais quand nous prenons en compte les autres variables (attitude, niveau de connaissance et soutien du conjoint), ce rôle explicatif n'apparait plus statistiquement significatif. En fin, les femmes qui avaient le soutien de leurs partenaire utilisaient les méthodes plus que celles qui n'en avaient pas (OR=5,7; IC95% 3,09-10,66), cela même quant nous considérons les autres variables prédictrices de l'utilisation des méthodes. (ORa= 3,42; IC95%: 1,82-6,45).

**Tableau 1:** Possession de l'information sur les méthodes contraceptives

Possession de l'information	Fréquence	Pourcentage
Non	45	9,0
Oui	455	91,0
Total	500	100

**Tableau 2:** Sources d'information sur les méthodes contraceptives

Sources	% (n=455)
Amis	13,6
Formations sanitaires	75,6
Eglise	13,8
Radio/télé	12,5

**Tableau 3:** Pévalence par méthode

Méthodes utilisées	Fréquence	Pourcentage
Préservatif masculin	88	17,6
Pilule	25	5,0
DIU	5	1,0
Depo provera	12	2,4
Implant	8	1,6
Méthode d'allaitement maternel et d'aménorrhée (MAMA)	32	6,4
Abstinence périodique	144	28,8
coïts interrompus	8	1,6
Aucune	178	35,6
Total	500	100

**Tableau 4:** Prévalence par catégorie des méthodes

Méthodes	Fréquence	Pourcentage
Modernes	138	27,6
Traditionnelles/Naturelles	184	36,8
Aucune	178	35,6
Total	500	100

**Tableau 5:** Ajustement des facteurs associés à l'utilisation des méthodes contraceptives

Variables	Utilisation MC (n=455)		OR brut (IC95%)	OR Ajusté (IC95%)
	Oui (%)	Non (%)		
**Age**				
15-19	5 (25,0)	15 (75,0)	1	NS
20-24	32 (27,8)	83 (72,2)	1,16 (0,35- 4,00)	
25-29	50 (35,7)	90 (64,3)	1,67 (0,52- 5,61)	
30-34	39 (33,3)	78 (66,7)	1,50 (0,46- 5,13)	
35 et +	12 (18,2)	51 (81,8)	0,71 (0,19- 2,74)	
**Statut matrimonial**				
Monogame	132 (30,3)	303 (69,7)	1,02 (0,36- 3,04)	NS
Polygame	6 (30,0)	14 (70,0)	1	
**Niveau d’étude**				
Primaire	14(28,0)	36 (72,0)	1	NS
Secondaire	88(31,1)	195 (68,9)	1,16 (0,57- 2,39)	
Universitaire	36 (29,5)	86 (70,5)	1,08 (0,49- 2,38)	
**Religion**				
catholique	63 (34,4)	120 (65,6)	1,78 (0,89- 3,61)	NS
Réformé/Eveil	52 (28,7)	129 (71,3)	1,37 (0,68- 2,80)	
Kimbanguiste	8 (32,0)	17 (68,0)	1,60 (0,51- 4,95)	
Autres	15 (22,7)	51(77,3)	1	

NS=non significatif

**Tableau 6:** Ajustement des facteurs associés à l'utilisation des méthodes contraceptive (suite)

Variable	Utilisation MC (n=455)		OR brut (IC 95%)	OR ajusté (IC95%)
	Oui	Non		
**Occupation**				
Ménage	71 (27,1)	191 (72,9)	1	NS
Libérale	57 (37,5)	95 (62,5)	1,61 (1,03- 2,53)	
Employée	10 (24,4)	31 (75,6)	0,87 (0,38- 1,96)	
**Confort du ménage**				
très bas	7 (27,9)	19 (73,1)	1	NS
bas	23(31,9)	49 (68,1)	1,27 (0,43- 3,90)	
moyen	102 (30,1)	237 (69,9)	1,17 (0,45- 3,17)	
élevé	6 (33,3)	12 (66,7)	1,36 (0,30- 6,08)	
**Nombre d'enfants vivants**				
0-2	87 (33,5)	173 (66,5)	1	NS
3-5	46 (28,6)	115 (71,4)	0,80 (0,51- 1,25)	
6 et +	5 (14,7)	29 (85,3)	0,34 (0,11- 0, 99)	
**Attitude de la femme**				
favorable	134 (35,6)	242 (64,4)	10,38(3,55-34,14)+++	4,79 (1,59-14,43)+++
Défavorable	4 (5,1)	75 (94,9)	1	1

NS=non significatif;

+++ = p<0,001

**Tableau 7:** Ajustement des facteurs associés à l'utilisation des méthodes contraceptive (suite et fin)

Variables	Utilisation MC (455)		OR brut (IC 95%)	OR ajusté (IC95%)
	Oui	Non		
**Niveau de connaissance des méthodes**				
Faible	59 (24,0)	187 (76,0)	1	1
Elevé	79(37,8)	130 (62,2)	1,93 (1,26- 2,95)***	1,87 (1,22-2,87)***
**Discussion avec le conjoint**				
couramment	82 (44,1)	104 (55,9)	6,23 (2,91- 13,69)***	0,74 (0,25-2,20)
Rarement	46 (25,6)	134 (74,4)	2,67 (1,22- 6,00)**	0,44 (0,15-1,23)***
jamais	10 (11,2)	79 (88,8)	1	1
**Soutien du conjoint**				
Oui	123 (39,9)	187 (60,3)	5,7 (3,09-10,66)***	3,42(1,82-6,45)***
Non	15 (10,3)	130 (89,7)	1	1
**Niveau d’étude du conjoint**				
Prim/sec	49 (27,5)	129 (72,5)	1	
Universitaire	89 (32,1)	188 (67,9)	1,25 (0,81- 1,93)	NS

NS=non significatif;

*** = p<0,001;

** =0,01

## Discussion

Nous avons observé que 91% des femmes étaient informées sur l'existence des méthodes contraceptives. Ces résultats plébiscitent les acteurs du domaine de sensibilisation en matière de planification familiale, car le message diffusé atteint une cible très importante. Les formations sanitaires étaient la source importante de cette information (75,6%); ces résultats sont différents de ceux obtenus dans la ville de Goba town en Ethiopie, qui montraient que les médias (radio et télévision) étaient la source principale d'information (87,3%) sur les méthodes contraceptives à longue durée d'action [12]. Cette différence tiendrait au fait que, dans notre milieu, la majorité de femmes utilisent le service de santé, soit pour les consultation pré natales (CPN), l'accouchement, la consultation pré scolaire (CPS) ou la consultation post natale (CPON), ce qui augmente la chance de recevoir l'information sur la contraception par ce canal; c'est aussi un signe que l'activité de planification familiale est intégrée dans les services de santé, car toute femme en union, a la chance de s'y rendre à des différentes circonstances inhérente à sa santé, en contradiction avec les médias dont le phénomène délestage prive à celles qui désirent les occasions de suivre les informations. Notre étude a révélé que 100% de femmes informées sur la contraception connaissent au moins une méthode contraceptive (moderne et/ou traditionnelle). Ce niveau élevé de connaissance a été observé dans les EDS-RDC de 2007: 91,1% des femmes en union dans le milieu urbain connaissaient au moins une méthode; dans la ville de Kinshasa, cette proportion s’élevait à 99,7% [4]. Ceci témoigne de l'efficacité des campagnes de sensibilisation sur les contraceptifs, autant par les structures sanitaires, que par d'autres moyens de communication de masses.

Quant à l'utilisation des méthodes contraceptives, la prévalence contraceptive moderne était de 27,6%. Ces résultats sont différents de ceux obtenus dans les EDS-RDC II 2013-2014 qui font état de 14,6% dans le milieu urbain [6]. Cette différence s'expliquerait par le fait que nos résultats n'ont concerné qu'une de 11 Zones de santé du district sanitaire de Lubumbashi, tandis que les EDS avaient tenu compte de toutes les villes de la RD Congo. Les résultats ont montré que les femmes qui avaient une attitude favorables vis-à-vis des méthodes contraceptives, avaient cinq fois plus de chances de les utiliser que celles qui avaient une attitude défavorable (ORa= 4,79; IC95%: 1,59-14,43, p<0,001). Ces résultats corroborent avec ceux obtenus au Bourkina-Faso, qui montraient que l'opinion favorable de la femme sur la planification familiale était significativement associée à l'utilisation des méthodes contraceptives modernes (p<0,001) [13] et ceux obtenus en Zambie, révélant que les femmes qui avaient l'attitude favorable avaient plus de chance d'utiliser la contraception moderne: OR=5,87; IC95%: 3,37-10,24 [14]. Ceci étant, une approche multidisciplinaire s'impose pour modeler les attitudes des femmes en union de la Zone de Santé Mumbunda, en relation avec une pratique contraceptive adéquate; car favorables seront leurs attitudes, hausse serait la prévalence contraceptive moderne. La notion d'attitude étant plus exploitée en psychologie sociale, le recours à ce domaine s'avère indispensable pour forger les attitudes favorables afin de booster l'utilisation des méthodes contraceptives modernes. Dans ces efforts, l'accent devra être mis sur la peur des effets secondaires, les rumeurs sur l'irréversibilité de la contraception etc., selon les résultats obtenus par Sedgh et al. (2007), relayés par Najafi-sharjobad F. et alliés [15]. Les femmes qui avaient un niveau élevé de connaissance des méthodes contraceptives avaient près de deux fois plus la chance d’être utilisatrices que celles qui avaient un niveau faible de connaissance (ORa=1,87, IC95%: 1,22-2,87, p<0,001). Ces résultats sont superposables à ceux trouvés en Ethiopie, montant que l'utilisation des méthodes contraceptives à longue durée d'action était associée à une connaissance modérée (ORa= 5,9; IC95%: 2,3-14,9) et à une connaissance élevée (ORa= 7,5; IC95%: 3,1-18,3) de ces méthodes [16]et ceux trouvés au Kenya, indiquant une association nette entre la connaissance des méthodes contraceptive et leur utilisation par les femmes (OR=1,06 IC: 1,03-1,08; p<001) [17]. La tendance était la même en Zambie, postulent les mêmes auteurs. C'est ici la nécessité de vulgariser les méthodes contraceptives à toute la population, en brisant toutes les barrières ou obstacles de la communication, car mieux les méthodes seront connues, élevée serait leur utilisation.

Près de la moitié (44,1%) des femmes qui discutaient couramment sur les méthodes contraceptives en utilisaient, suivis de 25,6% de femmes qui en discutaient rarement, contre 11,2% des femmes qui n'en discutaient jamais. Nous avons quantitativement observé que les femmes qui discutaient couramment et rarement avec leurs partenaires, avaient respectivement six (OR=6,23; IC95%: 2,91-13, 69) et trois (OR= 2,67; IC95%= 1,22- 6,00) fois plus la chance d'utiliser les contraceptifs modernes que celles qui ne discutaient jamais. En outre, cette association disparait après ajustement, ce qui lui confère un rôle confondant. Ces résultats ne corroborent pas avec ceux des différents auteurs: au Bourki-nafaso cette association a été significative: p<0,001 [13]. Takele et collaborateurs avaient trouvé que les femmes qui discutaient couramment avec leurs conjoints, utilisaient plus les méthodes que celles qui ne discutaient jamais (ORa=4.57; IC95%: 1.72, 12.17); cette association était inverse là où les discussions se faisaient une à deux fois (ORa=1) [12]. Dans le district de Butajira en Ethiopie, il a été trouvé également que les femmes qui avaient de discussion avec leurs partenaires sur la contraception, avaient près de trois fois plus la chance d'en faire usage que celles qui ne discutaient pas (ORa=2,6; IC95%: 1,8-2,7) [18]. Cette différence pourrait s'expliquer par le fait que discuter peut déboucher sur l'accord ou sur le désaccord avec le conjoint. Toute fois, le dialogue sur la contraception apporterait les effets bénéfiques à toutes les femmes qui en établiraient avec leurs conjoints, car le soutien ou l'encouragement du conjoint ne peut être obtenu qu'après une discussion. Au demeurant, les femmes qui avaient le soutien de leurs partenaires utilisaient les méthodes plus que celles qui n'en avaient pas (ORa=1,87, IC95%: 1,22-2,87, p<0,001). L’étude effectuée en Ethiopie, avait montré que les femmes qui avaient le soutient de leur mari avaient plus de deux fois la chance d'utiliser la contraception que leurs paires qui n'en bénéficiaient pas (ORa= 2,59; IC95%: 2,11-3,17 versus ORa=1) [18]; de même, Akelo V et Al (2013), avaient trouvé une association significative (p<0,001) entre le recours à la contraception et l'approbation du partenaire [19]. Ces résultats prouvent à suffisance le rôle que joue l'homme en matière de planification familiale. Etant donné que c'est l'homme qui décide sur beaucoup d'affaires courantes dans la vie conjugale, son implication est donc capitale si l'on désire améliorer effectivement la prévalence contraceptive; de même l'homme ne peut être écarté en planification familiale et s'attendre à des résultats meilleurs. Ainsi donc, les messages responsabilisant l'homme la planification doivent être plus fréquents et bien calibrés. Nous n'estimons par avoir pris en compte tout l'univers des déterminants, surtout ceux liés à l'organisation de service de planification familiale. Une nouvelle étude pourrait bien nous compléter, en privilégiant l'approche qualitative.

## Conclusion

Tout effort d'augmentation de la prévalence contraceptive, devrait cibler ces facteurs (attitude, connaissance de méthodes et le soutien du conjoint) afin d'optimiser l'atteinte de cet objectif. Ainsi, nous recommandons à tous les acteurs de la planification familiale de la zone de santé Mumbunda et tous ceux d'ailleurs, désirant améliorer la prévalence contraceptive dans cette entité, de promouvoir ces déterminants afin de booster l'utilisation des méthodes contraceptives modernes.
